# Rapidly established telehealth care for blood cancer patients in Nepal during the COVID-19 pandemic using the free app Viber

**DOI:** 10.3332/ecancer.2020.ed104

**Published:** 2020-09-02

**Authors:** Bishesh Sharma Poudyal, Bishal Gyawali, Damiano Rondelli

**Affiliations:** 1 Clinical Hematology and Bone Marrow Transplant Unit, Civil Service Hospital, Kathmandu, Nepal; 2 Departments of Oncology and Public Health Sciences, Queen’s University, Kingston, Canada; 3 Division of Cancer Care and Epidemiology, Cancer Research Institute, Queen’s University, Kingston, Canada; 4 Division of Hematology/Oncology, Department of Medicine, University of Illinois at Chicago, Chicago, IL, USA; 5 Center for Global health, University of Illinois at Chicago, Chicago, IL, USA

**Keywords:** Viber, COVID-19, haematological malignancies, chemotherapy

## Abstract

National lockdown to control the spread of COVID-19 in Nepal started in March 2020. This lockdown mandated closure of private and public transportation. The patients with hematological malignancies were at risk of delayed consultation, admission and missing scheduled chemotherapy. Since there is no official tele-health or e-health system established in hospitals, we decided to use Viber, a free text and call app to trace and provide information about patient admission and treatment schedule. This use of Viber during the pandemic was found to be very helpful, none of the patients missed chemotherapy and we were able to admit more patients than before. Patients found this strategy very convenient and cost-effective and suggested that we continue this service in future even after the lockdown is lifted. This preliminary experience of using Viber for cancer care consultations in Nepal at the time of the COVID-19 pandemic suggests the utility and acceptability of using mobile technology to improve access to health care services in a low-income country. Further pre-planned well conducted studies are needed to assess the outcomes of using this technology.

Nepal is a low-income country in South Asia, sandwiched between India and China, with an increasing cancer burden [1]. Health care in Nepal is not electronic yet, and use of electronic health records or tele-consultation is out of the scope of the country’s limited resources. However, the coronavirus pandemic forced us to adapt to the changing circumstances and we had to make do (known locally as the *jugaad* culture) [2] with the available resources to deliver care during the pandemic. Herein, we describe our preliminary experiences in Nepal with the use of Viber, a free call and text app for smartphones, as an example of the adoption of technology in the setting of a Low Income Country (LIC) during the pandemic.

In Nepal, the first COVID-19 case was diagnosed in January 2020 [3] and a national lockdown was imposed by the government in March 2020 [4]. The lockdown mandated closure of even public transportation upon which almost half of the Nepali population relies [5]. As a result, the usually overcrowded and only hospital providing comprehensive hematology services in Nepal, Civil Service Hospital, Kathmandu, become almost empty of patients.

This lockdown and closure of public transport made physicians suspend clinical visits, including for patients with severe blood malignancies, such as leukemias and lymphomas. Since no official tele-health, or e-health systems have been established in the country, the only hematologist and bone marrow transplant physician of the hospital (the first author of this paper) decided to use Viber, a free text and call app (similar to Whatsapp) as a substitute for formal e-consultation software. Many of his patients were already aware of this app since it is commonly used in Nepal for personal chat and calls [6].

The physician’s Viber phone number was placed on the hospital’s website, posted on social media and distributed to each follow-up patient via phone call. Since people are forbidden to go out of the house during the lockdown and would face police custody for violation, patients who needed to commute to the hospital for chemotherapy would show police officers the Viber texts received from the physician along with their hospital card, to receive permission for travel during the lockdown. Although no formal arrangement with the police department was made, the majority of the patients were able to travel during the lockdown using this strategy.

By day 40, more than 1500 messages from 333 patients had been answered. The majority of these messages were from patients with acute leukemia, both newly diagnosed and established patients. Patients could ask questions via Viber texts and receive an answer quickly. Based on the details of the initial message sent by the patient, the physician would explain the diagnosis and/or chemotherapy schedules through return messages, or would request the patient to undergo further laboratory or radiologic investigations at a local nearby hospital. The results of these tests would be then texted by patient to the physician and the physician would recommend dose modifications, further investigations, changes in treatment, or general counseling. If there was an immediate need to examine the patient, travel to hospital was managed accordingly through the emergency services.

None of the established patients managed via Viber missed chemotherapy. Patients, instead, found this strategy helpful because they were able to consult with the treating physician very easily. The majority of the patients suggested that the Civil Service Hospital providers continue this service even after the lockdown ends in the future. Many patients also informed us that this method of consultation was very convenient and cost-effective since it saved their travel and accommodation expenses if they had to travel to Kathmandu. In fact, since Civil Service Hospital has the only comprehensive Haematology/BMT department in Nepal, patients often commute from outside Kathmandu for many hours or days. During these months more newly diagnosed patients were admitted to the hospital compared to the past (around 50 inpatients a day now versus 24 before the pandemic). A possible explanation seems to be the ease of communication with the physician. Before COVID-19, patients stood in a queue for 2-3 hours just to book an appointment. Furthermore, because the new patient consults in our outpatient haematology clinic are restricted to 30 patients per day (with no restriction on number of follow up patients), many patients would be denied an appointment. Now, using Viber free text messaging, it was possible to receive medical attention on the same day irrespective of one’s queue number and a direct admission to the hospital would be arranged when necessary. The second explanation is that although routinely the haematology unit has only 24 inpatient beds, currently there are enough empty beds in the hospital in other units that we could use.

This experience reveals multiple opportunities for an LIC such as Nepal, where access to cancer treatment is limited due to the small number of specialized centres. Using existing apps such as Viber resulted in a fast and direct contact between patient and physician; efficiency in hospital admission for newly diagnosed patients or scheduling chemotherapy; and transportation for patients who often live far from Kathmandu.

We are aware of the limitation that Viber is not Health Insurance Portability and Accountability Act (HIPAA) compliant. However it is secured by end-to-end encryption, which makes communication across different devices safe and secure. Furthermore, the data are shared directly and voluntarily by patients to physicians. Although HIPAA compliant live chat apps are available, Viber is already used by the majority of the population regardless of social status or technologic education and does not need any training. In an emergency circumstance, such as a national disaster or a pandemic, the timing for implementing an accessible supportive plan is a top priority element. In a study conducted in Italy, WhatsApp (an app similar to Viber) was found to be effective to give a rapid answer to most queries from patients with cancer in the COVID-19 pandemic scenario [7].

This editorial highlights our preliminary experience during the pandemic. It was not a pre-planned study, and therefore our experience does not provide any definitive conclusions but only suggests that such an approach was well accepted by patients and could be studied more formally in the future.

In conclusion, the experience of using Viber for hematological malignancies at the time of the COVID-19 pandemic demonstrates the feasibility of using accessible technology to improve health care services in a country with limited economic resources and healthcare infrastructures like Nepal.

## Conflicts of Interest

None

## Author's contribution statement

BSP designed the research, collected and analyzed the data. BSP, DR and BG wrote the manuscript and revised the manuscript. All authors agreed to the final manuscript and its submission. Patients described in this study were primarily treated under BSP’s care.
